# Supplementary material for: Group structure and individual relationships of sanctuary-living Grauer’s gorillas (Gorilla beringei graueri)
Source: PLoS One. 2024 Jan 17;19(1):e0295561. doi: 10.1371/journal.pone.0295561 (PMC10793932; doi:10.1371/journal.pone.0295561)
Supplement: S1 File — This word document contains three tables containing the association index for each pair by observation context (S1, mixing; S2, shifting; S3, nesting). (DOCX) [file pone.0295561.s002.docx]

**Table S1**. Association indices for each gorilla pair from mixing yard observations.

|  | **AMA** | **ISA** | **ITE** | **KAL** | **KIG** | **LUB** | **LUL** | **MAP** | **MUY** | **NDJ** | **PIN** | **SER** | **SHA** | **TUM** |
| --- | --- | --- | --- | --- | --- | --- | --- | --- | --- | --- | --- | --- | --- | --- |
| **AMA** | - | 0.12 | 0.04 | 0.06 | 0.09 | 0.03 | 0.09 | 0.26 | 0.11 | 0.04 | 0.02 | 0.01 | 0.03 | 0.06 |
| **ISA** | 0.12 | - | 0.03 | 0.03 | 0.02 | 0.01 | 0.05 | 0.03 | 0.05 | 0.03 | 0.03 | 0.05 | 0.04 | 0.02 |
| **ITE** | 0.04 | 0.03 | - | 0.04 | 0.06 | 0.03 | 0.01 | 0.03 | 0.08 | 0.03 | 0.03 | 0.03 | 0.05 | 0.08 |
| **KAL** | 0.06 | 0.03 | 0.04 | - | 0.13 | 0.01 | 0.05 | 0.05 | 0.04 | 0.03 | 0.03 | 0.04 | 0.05 | 0.03 |
| **KIG** | 0.09 | 0.02 | 0.06 | 0.13 | - | 0.04 | 0.07 | 0.08 | 0.17 | 0.08 | 0.01 | 0.05 | 0.07 | 0.04 |
| **LUB** | 0.03 | 0.01 | 0.03 | 0.01 | 0.04 | - | 0.01 | 0.03 | 0.03 | 0.03 | 0.01 | 0.04 | 0.05 | 0.02 |
| **LUL** | 0.09 | 0.05 | 0.01 | 0.05 | 0.07 | 0.01 | - | 0.05 | 0.05 | 0.04 | 0.12 | 0.02 | 0.04 | 0.03 |
| **MAP** | 0.26 | 0.03 | 0.03 | 0.05 | 0.08 | 0.03 | 0.05 | - | 0.06 | 0.04 | 0.07 | 0.03 | 0.03 | 0.03 |
| **MUY** | 0.11 | 0.05 | 0.08 | 0.04 | 0.17 | 0.03 | 0.05 | 0.06 | - | 0.17 | 0.03 | 0.03 | 0.07 | 0.07 |
| **NDJ** | 0.04 | 0.03 | 0.03 | 0.03 | 0.08 | 0.03 | 0.04 | 0.04 | 0.17 | - | 0.02 | 0.03 | 0.04 | 0.04 |
| **PIN** | 0.02 | 0.03 | 0.03 | 0.03 | 0.01 | 0.01 | 0.12 | 0.07 | 0.03 | 0.02 | - | 0.08 | 0.06 | 0.08 |
| **SER** | 0.01 | 0.05 | 0.03 | 0.04 | 0.05 | 0.04 | 0.02 | 0.03 | 0.03 | 0.03 | 0.08 | - | 0.01 | 0.02 |
| **SHA** | 0.03 | 0.04 | 0.05 | 0.05 | 0.07 | 0.05 | 0.04 | 0.03 | 0.07 | 0.04 | 0.06 | 0.01 | - | 0.03 |
| **TUM** | 0.06 | 0.02 | 0.08 | 0.03 | 0.04 | 0.02 | 0.03 | 0.03 | 0.07 | 0.04 | 0.08 | 0.02 | 0.03 | - |

**Table S2**. Association indices for each gorilla pair from shifting observations.

|  | **AMA** | **ISA** | **ITE** | **KAL** | **KIG** | **LUB** | **LUL** | **MAP** | **MUY** | **NDJ** | **PIN** | **SER** | **SHA** | **TUM** |
| --- | --- | --- | --- | --- | --- | --- | --- | --- | --- | --- | --- | --- | --- | --- |
| **AMA** | - | 0.23 | 0.1 | 0.2 | 0.12 | 0.08 | 0.05 | 0.27 | 0.13 | 0.14 | 0.05 | 0.02 | 0.07 | 0.06 |
| **ISA** | 0.23 | - | 0.04 | 0.03 | 0 | 0.02 | 0.01 | 0.04 | 0.05 | 0.03 | 0.03 | 0.01 | 0.03 | 0.03 |
| **ITE** | 0.1 | 0.04 | - | 0.03 | 0.01 | 0.01 | 0.04 | 0.03 | 0.02 | 0.03 | 0.03 | 0.02 | 0.01 | 0.01 |
| **KAL** | 0.2 | 0.03 | 0.03 | - | 0.19 | 0.02 | 0.02 | 0.04 | 0.06 | 0.06 | 0.06 | 0.01 | 0.06 | 0.02 |
| **KIG** | 0.12 | 0 | 0.01 | 0.19 | - | 0.03 | 0.02 | 0.05 | 0.05 | 0.04 | 0.03 | 0.01 | 0.06 | 0.01 |
| **LUB** | 0.08 | 0.02 | 0.01 | 0.02 | 0.03 | - | 0.01 | 0.03 | 0.03 | 0.03 | 0.03 | 0.04 | 0.07 | 0.01 |
| **LUL** | 0.05 | 0.01 | 0.04 | 0.02 | 0.02 | 0.01 | - | 0.03 | 0.04 | 0.03 | 0.09 | 0.05 | 0.06 | 0.04 |
| **MAP** | 0.27 | 0.04 | 0.03 | 0.04 | 0.05 | 0.03 | 0.03 | - | 0.06 | 0.05 | 0.07 | 0.03 | 0.04 | 0.02 |
| **MUY** | 0.13 | 0.05 | 0.02 | 0.06 | 0.05 | 0.03 | 0.04 | 0.06 | - | 0.2 | 0.01 | 0.03 | 0.05 | 0.04 |
| **NDJ** | 0.14 | 0.03 | 0.03 | 0.06 | 0.04 | 0.03 | 0.03 | 0.05 | 0.2 | - | 0.05 | 0.02 | 0.05 | 0.01 |
| **PIN** | 0.05 | 0.03 | 0.03 | 0.06 | 0.03 | 0.03 | 0.09 | 0.07 | 0.01 | 0.05 | - | 0.13 | 0.1 | 0.08 |
| **SER** | 0.02 | 0.01 | 0.02 | 0.01 | 0.01 | 0.04 | 0.05 | 0.03 | 0.03 | 0.02 | 0.13 | - | 0.08 | 0.03 |
| **SHA** | 0.07 | 0.03 | 0.01 | 0.06 | 0.06 | 0.07 | 0.06 | 0.04 | 0.05 | 0.05 | 0.1 | 0.08 | - | 0.04 |
| **TUM** | 0.06 | 0.03 | 0.01 | 0.02 | 0.01 | 0.01 | 0.04 | 0.02 | 0.04 | 0.01 | 0.08 | 0.03 | 0.04 | - |

**Table S3**. Association indices for each gorilla pair from nesting observations.

|  | **AMA** | **ISA** | **ITE** | **KAL** | **KIG** | **LUB** | **LUL** | **MAP** | **MUY** | **NDJ** | **PIN** | **SER** | **SHA** | **TUM** |
| --- | --- | --- | --- | --- | --- | --- | --- | --- | --- | --- | --- | --- | --- | --- |
| **AMA** | - | 0.19 | 0.13 | 0.11 | 0.13 | 0.08 | 0.04 | 0.52 | 0.1 | 0.18 | 0.06 | 0.02 | 0.06 | 0.14 |
| **ISA** | 0.19 | - | 0.06 | 0.04 | 0.06 | 0.05 | 0.03 | 0.05 | 0.06 | 0.13 | 0.04 | 0.02 | 0.07 | 0.11 |
| **ITE** | 0.13 | 0.06 | - | 0.04 | 0.01 | 0.03 | 0.01 | 0.07 | 0.05 | 0.04 | 0.03 | 0.02 | 0.03 | 0.06 |
| **KAL** | 0.11 | 0.04 | 0.04 | - | 0.53 | 0.01 | 0.02 | 0.04 | 0.13 | 0.01 | 0.03 | 0.01 | 0.04 | 0.05 |
| **KIG** | 0.13 | 0.06 | 0.01 | 0.53 | - | 0 | 0.01 | 0.01 | 0.16 | 0.02 | 0.01 | 0.01 | 0.04 | 0.02 |
| **LUB** | 0.08 | 0.05 | 0.03 | 0.01 | 0 | - | 0.03 | 0.02 | 0.06 | 0.06 | 0.04 | 0.1 | 0.29 | 0.03 |
| **LUL** | 0.04 | 0.03 | 0.01 | 0.02 | 0.01 | 0.03 | - | 0.02 | 0.01 | 0.02 | 0.62 | 0.12 | 0.01 | 0 |
| **MAP** | 0.52 | 0.05 | 0.07 | 0.04 | 0.01 | 0.02 | 0.02 | - | 0.06 | 0.04 | 0.04 | 0.03 | 0.01 | 0.06 |
| **MUY** | 0.1 | 0.06 | 0.05 | 0.13 | 0.16 | 0.06 | 0.01 | 0.06 | - | 0.19 | 0.01 | 0.02 | 0.06 | 0.05 |
| **NDJ** | 0.18 | 0.13 | 0.04 | 0.01 | 0.02 | 0.06 | 0.02 | 0.04 | 0.19 | - | 0.03 | 0.02 | 0.07 | 0.07 |
| **PIN** | 0.06 | 0.04 | 0.03 | 0.03 | 0.01 | 0.04 | 0.62 | 0.04 | 0.01 | 0.03 | - | 0.4 | 0.08 | 0.04 |
| **SER** | 0.02 | 0.02 | 0.02 | 0.01 | 0.01 | 0.1 | 0.12 | 0.03 | 0.02 | 0.02 | 0.4 | - | 0.01 | 0.03 |
| **SHA** | 0.06 | 0.07 | 0.03 | 0.04 | 0.04 | 0.29 | 0.01 | 0.01 | 0.06 | 0.07 | 0.08 | 0.01 | - | 0.05 |
| **TUM** | 0.14 | 0.11 | 0.06 | 0.05 | 0.02 | 0.03 | 0 | 0.06 | 0.05 | 0.07 | 0.04 | 0.03 | 0.05 | - |
