# Supplementary material for: Group structure and individual relationships of sanctuary-living Grauer’s gorillas (Gorilla beringei graueri)
Source: PLoS One. 2024 Jan 17;19(1):e0295561. doi: 10.1371/journal.pone.0295561 (PMC10793932; doi:10.1371/journal.pone.0295561)
Supplement: Attachment — Submitted filename: Response to Reviewers.docx [file pone.0295561.s003.docx]

| **Editor Comment(s)** | **Author Response** |
| --- | --- |
| I have some suggestions for your manuscript that you might consider. Additionally, the conclusions section was difficult to understand, so please revise it to be more clear and concise. I think these attached comments will be very helpful in the revision of your manuscript. | Thank you. We have addressed each reviewer’s comments/edits and have incorporated nearly every suggestion. We have additionally updated the wording of the conclusion section and hope it is now clearer in its content and presentation. |

| **Reviewer 1 Comments** | **Author Response** |
| --- | --- |
| 3. Have the authors made all data underlying the findings in their manuscript fully available?  - **Reviewer 1: No**  - Reviewer 2: Yes | The full dataset used in the analysis is included as supplementary information. Are there concerns with the data’s presentation? We are happy to correct if advised on a specific concern. |
| Before anything, I will acknowledge that I have limited experience with research on captive animals, so some of my suggestions are just that: suggestions. But I do have a few concerns that go beyond the suggestion part, and to which I would prefer to receive a response (or see edits/changes). | Thank you. We have taken all of your edits/changes into consideration and have incorporated them into the revised manuscript. |
| Overall, I think that there are some minor things to adjust and then this can be a good contribution. But, following the authors own words (Discussion), I 'do' think the whole emphasis of the paper should lie a bit more on the application for captive management part, and a bit less on the real-world ecological consequences. | Thank you. We agree more emphasis on the applied would benefit this manuscript. See below for additional comments but we have increased emphasis on applied value, reduced focus on comparisons to populations in nature, and when we do compare to nature we use conservative language highlighting the data/comparisons are “tentative”, “preliminary” and/or “provisional” in the absence of more detailed description of Grauer’s gorilla behavior in nature. We have also shifted the order of our abstract and conclusion to discuss applied values first and reference comparisons to nature last. |
| First, I am wondering if the whole phrasing of the Introduction, and the general premise for your ‘story’, should not be flipped. Right now, the focus lies heavily on the insights on nature of Grauer’s gorilla social networks themselves (that we can extrapolate from this captive population to the ‘wild’ etc.) and less on the captivity/management part. I would think that, like other studies on zoo/captive populations, this study mainly has value as a work to inform the management of captive Grauer’s’ and provides only tentative insights in the natural history of the ‘wild’ species (more like suggestions for further research, areas where these behaviors differ or confirm what is known from populations of other subspecies). In short: I think you might want to do a little reframing: focus on the applied benefits foremost and only gently make inferences for the larger ecological/evolutionary relevance of your study. EDIT (after reading on): basically like you propose in lines 118-120, focus on paper on objectives 1 and 2 and only briefly touch upon what this could mean for objective 3. | Thank you for this helpful suggestion.  We have reworked our introduction to fit this suggestion. This required significant reorganization/rewriting so we apologize we cannot provide specific line by line changes (and also apologize the track changes version is now a mess) but broadly we made the following changes:   - Reworded the the opening paragraph to emphasize applied value of this line of research (broadly speaking). - Significantly reduced the paragraphs focused on gorilla natural history/social structure to two concise paragraphs. We did not want to eliminate these entirely but feel our heavy reduction of these sections allowed for more space to discuss applied purposes and minimizes the emphasis on comparing directly to natural history. - We significantly expanded the paragraph describing our three objectives you highlighted (previously line 118-120). - To the above point - we maintained objective 3, as we do feel there is biological value in studying them in this way but have softened our language – mirroring your suggestion and used the phrase “… may provide tentative insights into…” We feel the inclusion of the words “may” and “tentative”, as well as lengthening the discussion of the first two objectives have reduced the emphasis on this third objective. - While not specified here, the “flipping” of the introduction required some changes to our discussion. These are more clear from the track changes as we did not do a complete overhaul in the writing but attempted to include applied perspectives to our discussion topics where relevant, or expand on applied interpretations to make them the main point of emphasis.   - Furthermore, the conclusion has strong language as well that we think balances the value of such comparisons with the caution it needs. Now reads:     - “Lastly, in the absence of detailed information from populations in nature this study provides provisional, but important, insights into the social structure of Grauer’s gorillas. Though not a replacement or equal in value to findings from in-situ field research, this study adds to a growing body of informative research conducted within in-situ primate sanctuaries [52,53].” |
| Second, it strikes me that in the paragraphs (Intro) on relationships you make no reference to the importance/our understanding of kinship/kin-specific relationships. That seems to be an integral part of the formation of intragroup associations and I’d think it needs to be mentioned more explicitly (early on in the Intro). See e.g., Morrison, R. E., Groenenberg, M., Breuer, T., Manguette, M. L., & Walsh, P. D. (2019). Hierarchical social modularity in gorillas. Proceedings of the Royal Society B, 286(1906), 20190681. | Thank you highlighting this missing component.  As we mention in a response to a similar comment/suggestion below, we were attempting to be concise but clearly missed some significant components. We have included this citation and the importance of kinship in social structure into the opening paragraph, second sentence. |
| This is a subjective style choice, but I personally think that an active voice would improve the flow of the manuscript. As far as I’m concerned, you can take or leave that suggestion, but I really think it could be beneficial to the overall readability. Again, no need to change it if you don’t want it! | We have switched the writing of the manuscript to the active voice. |
| 29: maybe just to avoid confusion: ‘animals living under/in human care’? | We have clarified and now reads “… and can inform management decisions for animals living in human care.” |
| 32: officially, I think it’s ‘of the Congo’ | Embarrassing error – corrected throughout. |
| 32: just a reminder that the Abstract should stand alone, and that the reader does not, at this point, yet know what ‘the GRACE group’ is (are the gorillas in the sanctuary housed together in one group? What does that group look like? Are we talking about 5 gorillas or 20?).  I see some of this throughout the abstract, just make sure that every sentence and every piece of information can only be clearly interpreted in one single way.  This sentence is also a perfect example of how an active voice could make it easier to read: “we monitored…..over the course of eight months….” | We have clarified two notable points in the abstract (noted below) and have switched to the active voice.  “…a group of 14….” was added to the second sentence introducing GRACE to provide additional detail.  “in three management contexts” was remove as this was unclear for abstract and would require too many words to properly contextualize in this space. |
| 32-37: we also don’t yet know what these contexts are, which makes it a bit hard to follow. | Agreed – as above, this was removed. |
| 40-43: Well, yes, but it is worth re-emphasizing that this is not a natural situation. Social network analyses on mountain gorillas might be more representative of naturally occurring dynamics than a social network found among a captive group. So, I would bring this with more caution. | As both comments relate to same sentence we have combined feedback here.  We split the sentence into two and removed/added a few key words making it more cautious as to the potential scope. Now reads:  “Lastly, our study is the first social network analysis to be conducted on Grauer’s gorillas and provides tentative insights into the behavior of this poorly studied subspecies. Though more research is needed to evaluate if the findings here are reflective of this subspecies’ natural history or the idiosyncrasies of the group” |
| 41: try to avoid long sentences (e.g., instead of continuing with “…, however,” just split it into two sentences). Especially given the fact that you use a passive voice, it becomes a bit lengthy here and there. |  |
| 56: is this complete (or even the most important ones)? What about demographic factors and kinship? Or do you see that as drivers ‘within’ fitness outcomes? I think it might need to be mentioned explicitly? | Agreed, in our attempt to be brief on a broad topic we cut out arguably the most important aspect of social structure – group demographics. We now state “… group demographic factors such as group size, sex ratios and kinship…” as contributing to group structure. |
| 59: reference maybe? | In the rewriting of the introduction this sentence was removed. |
| 67: as you rightly say in the next sentence, this is not the case for mountain gorillas, so isn’t this a bit of an odd statement? Maybe add some modifier such as ‘predominantly’ etc.? | In the rewriting of the introduction this sentence was removed as part of our reduction of natural history content in favor or applied content. |
| 69: I’m not really clear on the ‘by site/year’ part, what does that refer to? | In the rewriting of the introduction this sentence was removed as part of our reduction of natural history content in favor or applied content. |
| 78: be careful with words such as ‘prefer’ (they tend to do this, yes) | Replaced “prefer” with “tend” |
| 95-97: seems to bit of an odd juxtaposition to me. Sure, they are affected by disturbances, but its unlikely that the response of Grauer’s to human disturbances differs much from that of the other gorilla subspecies. | That is a fair point. What we were attempting to highlight here was a heavy round of poaching occurred within KBNP while Grauer’s gorillas were being actively studied (i.e. only study site for species, few study groups, many deaths) that likely influenced the “population” data more so than other subspecies that are studied longer term and at more sites.  Similar to above, in our efforts to focus on applied components of this study – this detail/sentence was removed. |
| 98: I would say nearly never but ok | Replaced “infrequently” with “rarely” |
| 99: population rate? | In the rewriting of the introduction this sentence was removed as part of our reduction of natural history content in favor or applied content. |
| 100: worth mentioning though that this was restricted to observations of one small population (4 groups) (of closely related gorillas). | In the rewriting of the introduction this sentence was removed as part of our reduction of natural history content in favor or applied content.  However, in expanding discussion of rerelease value in the discussion we did include this point. We have added the important qualifiers you highlight here. Sentences read:   - Line 437 “In Grauer’s gorillas, nearly half of female emigrations occur in groups, suggesting females may rely on social support during these significant life events [79]. However, this finding is from a single population of presumably closely related female gorillas and thus may be driven by group demographics not present in the GRACE group.” |
| 104-106: you do have a disclaimer (“more studies needed”) but even then, I find this a rather tentative hypothesis based on very little data. I don’t think you can say this at this point. Also, “these findings”, you mean those findings that “females with infants more time resting together” or all the findings in the previous sentences? | Sentence was removed. |
| 120-123: I think that it has to be clear early on that these gorillas are not (necessarily) kin/related, unlike a group in the wild (or even most individuals in zoo groups) | Added “…all of whom are presumed to be unrelated.” |
| 142: from wild populations (after all, even captive organisms are natural ) | Here we use the phrase “in nature” to mean “in the wild.” We prefer not to use the word “wild” because it (historically) has been used to imply “savageness/wildness” of non-human animals, particularly primates. We use the phrase ~10 times in the paper when referencing in-situ populations so we hope it is just the use in this particular sentence that stood out and not the entirety of this phrase’s use. We rephrased this sentence to improve flow as we acknowledge it was a bit choppy. The sentence now reads: LINE 134 “Within the above analyses we placed additional focus on the relationships of the three males within the group as this subspecies predominantly lives in single male groups in nature [35].” |
| 155: 3.11? you mean 3 males and 11 females for a total of 14? I don’t know if this short-hand description works for the broad audience of this journal; would suggest to write it out. | Yes the formatting translates to male#.female#. We use it commonly in human care, we have corrected. Now reads: “A group of 14 (n_male_ = 3; n_female_ = 11) Grauer’s gorillas…” |
| 247: Spearman test is base R, no need to call the ‘program’ Rcdmr, just state “…test using R [54]”. | Corrected |
| 259: Bonferroni | Corrected |
| 262-270: I think there are some details missing in this paragraph. What did the linear regression look like, e.g., in terms of data family? And the power regression? Power of two I assume …? Also when you say “regression fit statistics including…” then I’m thinking, which other ones? | We provide a response here for both this and the subsequent related comment.  Great questions, and yes you are correct some details were missing. We have corrected this by providing additional context in the manuscript, which is outlined below.  Following the methods of Bret et al (2013) and Ramos et al (2019) we fit a simple linear regression (y = a + bx) and a power regression (y = ax^b) to the data. Because these were basic regressions and not GLM type multiple regression no data family were specified. We have now included the regression equations in our methods description to specify how our calculations were completed.  The inclusion of the statement “regression fit statistics including” is a typo from a previous draft in which we included both AIC and R2 as fit measures. We choose to remove R2 as it was redundant to AIC. We apologize for the confusion. This has been corrected in manuscript.  Neither regression output included an AIC value by default thus we calculated AIC values by hand to compare regression fits against one another. The data were normally distributed (as assessed by shapiro-wilk test and visual inspection of QQ plots) and thus an AIC value calculated with residual sum of squares (i.e. Gaussian AIC) was selected as a measure of model fit strength. This detail was not included in original draft and is now included to justify our use of AIC.  Use of AICc is a potential option, however, this term is typically used to assess more complex models (models with multiple parameters) because AIC is more likely to over fit models with small sample sizes that also include multiple parameters. Given our use of simple regressions here this may not be the best option since it accounts for something not present in our model (i.e. multiple predictive parameters). We prefer to maintain our use of AIC given these considerations. |
| 266: is this Gaussian definition of the AIC the appropriate one? Please ignore my ignorance but it’s not the general definition (as used in e.g., Burnham, K. P.; Anderson, D. R. (2002), Model Selection and Multimodel Inference: A practical information-theoretic approach (2nd ed.), Springer-Verlag.) but an adapted one, and I’m wondering why. Also, might AICc (correction for small sample size) not be more appropriate? |  |
| 304: just a suggestion but does this all need to written out or can it be put into a table for readability? | We feel this component of the reporting needs to be written out. Here we took the AI values and described how individual pairs related to overall means of all 91 pairs. We felt this was a good summary of a larger dataset, highlighting important trends that is best explained by “talking out.” However, in considering how we may make this a table we realized we did not include tables listing each pairs AI/context (i.e. the data these summaries are made from). Since the data described here all relate back to these values we think it’s a mistake not to include them. We have now included three new tables listing each pairs AI value (one for each context). They are a bit large so have included them as supplementary tables labeled as Tables S1, S2 and S3. |

| **Reviewer 2 Comments** | **Author Response** |
| --- | --- |
| This study provides the first examination of Grauer’s gorilla social behavior in a sanctuary or captive setting. It provides a relatively straightforward and easily scalable social network approach to comparing behavioral association data collected on captive animals in different contexts. The authors provide a clear and succinct background explaining the context and overall aims for the study. They do a great job in detailing the analytical framework that was used. Overall, the paper is well-written, concise, and has important implications for sanctuary managers regarding the maintenance of long-term social associations among dyads of captive gorillas. I have only a few minor questions and suggestions for the authors and have provided detailed in-line comments below. | Thank you. We appreciate the feedback and feel strongly we have adequately incorporated your comments/suggestions/edits. |
| Line 155: Typo. Please clarify group size. | Corrected. See reviewer 1 comment for additional detail. |
| Line 162: Please indicate if females are on birth control. This is important biological information that may influence inter-individual associations in a sanctuary context. Also, please indicate if genetic data is available for these individuals. | - The females are on an estrogen/progestin combination birth control pill. We have added a new column to Table 1 labeled “Fertility Status,” this allows us to highlight the females are on contraceptives and the males are intact as part of the overall individual summary information. We additionally added a “developmental status” column to the table as we felt that further highlighted the reproductive status of the individuals. - Could you clarify what you mean by “are genetic data available?” We are not sure what specific information you are requesting. To provide a preliminary answer (and apologies if missing your point) - there are no available genetic samples to share for these individuals, nor has DNA testing been conducted on all individuals. We do state in the “study group” section that it is “presumed that none of the individuals are related” as this is the state of our knowledge on their relatedness. If the request is focused on a general statement about sample availability we do not think this is needed as genetic analysis does not relate to this study. |
| Line 172: Please be specific about what these “provided dietary items” were, whether gorillas were allowed to free forage on growing plants within the forest enclosure, and the spatial distribution of food items. If provisioned foods were clumped within the mixing yard or if gorillas were consuming plants in the forest enclosure that were spatially clumped, this may have affected feeding associations. | For each of your three questions:   - In the mixing yard the gorillas were provided forest vegetation included elephant grass and bramble (Rubus sp.). Additionally, fruit purchased from a local market is also provided when available. This information has been added to this section LINE 165. - Food items are spread throughout mixing yard and not clumped. This has been added LINE 167. - In the forest enclosure the gorillas were allowed to forage freely on growing plants. Plants grew freely and may be more or less clumped/spread out depending on a variety of factors. This information is added LINE 171. True distribution rates of food in these large multi acre enclosures is unknown.   Section now reads: “Gorillas at GRACE are housed in six different areas: two interconnected night houses composed of 10 individual rooms totaling approximately 210 m2 in size, an outdoor yard (mixing yard) approximately 3,000 m2 in size and two large forested enclosures approximately 81,600 m2 and 46,300 m2 in size. In a typical 24 hr period the gorillas spend the night in the night houses, choosing which room and which gorillas to be with. In the morning the gorillas are then moved to the mixing yard where they have access to provided diet items for approximately one hour. Provided dietary items include vegetation harvested from the forest enclosures (e.g. elephant grass, Rubus sp.) and fruit purchased from local markets (e.g. passion fruit, banana, pineapple, plum), all of which are spread throughout the mixing yard. The group then shifts to one of the two forest enclosures for the day. Forest enclosure access is provided in blocks of time such that gorillas have access to one enclosure for approximately six to nine months then have access to the alternative enclosure for a similar amount of time. During this study the gorillas had access to the smaller forest enclosure each day. Vegetation grows freely throughout both enclosures that the gorillas are free to forage on. Midday, the gorillas are recalled into the mixing yard, followed by a return to the forest enclosure. At the end of the day the gorillas are recalled back to the mixing yard and then to the night houses.” |
| Lines 178 – 190: It would be helpful if the authors justified why they chose an association threshold of 1 m. Also, was activity of gorillas noted during observations? (i.e., whether feeding, resting, or other?). If not, state this here in methods. Furthermore, the scan sampling protocol suggests more scan data were collected in the mixing yard context (3 scans per observation period) vs. in the nesting or forest enclosure setting (1 scan per observation period). This may affect interpretations of network correlation analyses that were described in Lines 255-260. How did the authors account for temporal autocorrelation for scans collected in the mixing yard context? | - We have included a justification for 1m in the manuscript. This reads as follows, LINE 179 “For each context, each gorilla dyad (91 total) was recorded as being associated (within 1 m) or not. Studies of gorilla associations in nature frequently utilize within 5m as their metric [29,55], however, recent research has highlighted closer metrics (within 2m) provide more definition in association measures [4]. We thus sought to adopt a similarly conservative approach, however, we utilized 1m as it provided a more reliably trained metric as it was approximately equal to an adult gorilla arm length. This visual cue aided researchers who at times observed the gorillas from distances greater than 25m.” - The activity of the gorillas was not noted. On LINE 180 we highlight that associations were the only data collected. - For the 3 scans in the mixing yard/autocorrelation. We conducted three scans but report it as a single data point per day. On LINE 211 we highlight this but have now added some additional verbiage to better describe our method. Now reads: “Mixing yard data were collected over three scans. To reduce concerns of autocorrelation between scans conducted in the same day we generated a single one/zero association score for all three scans (i.e. if a pair were associated in at least one of the three scans they were coded as associated for the mixing yard that day).” |
| Lines 190 – 193: The authors make an interesting observation that the gorillas tended to congregate towards the shift door prior to their recall. How did the authors account for spatial limitations across the three contexts? It is possible that the smaller night houses might have limited inter-individual spacing compared to the outdoor foraging contexts. Thus, sleeping associations may not have been an indication of preference but rather a spatial constraint. It would help to justify this choice. Also, it appears as if the authors do not distinguish between scans that were collected in one forest enclosure over the other. The authors note that the two forests enclosures are different sizes; are there also important differences in how plants are clumped in these two spaces? Some justification on why it was appropriate to lump these observations into one category would be helpful. | - To the first question on spatial limitations of three contexts. We analyzed each observation context separately because each context had different space availability, as well as other context specific factors that may have affected associations. Sentence now reads: LINE 223 “All analyses were conducted in SOCPROG Version 2.9 [57] unless stated otherwise and all analyses were conducted independently for each observation context to control for differences in space availability as well as additional factors independent to each context that may have influenced associations” - For the night house specifically. The night house was the smallest area the gorillas have access to. However, there is still sufficient room to spread out with 10 rooms (and 14 gorillas) and most (8/10) rooms are greater than 6m across at the shortest point. As each context was analyzed independently we can discuss each context’s findings in their own respective terms. To this point we added the following to the discussion LINE 502: “It is also surprising that association indices were correlated because of the notable differences in space availability in each respective context. Individuals generally associated in similar ways whether they were in the multi-acre forest enclosure or night house, further suggesting association indices were reflective of individual choices and not space constraints.” - For forest enclosures – during this study the gorillas only had access to one enclosure (they are rotated over 6-9 month periods). This has been clarified in the methods on line 168. |
| Line 263: Clarify how cumulative eigenvector centrality was calculated for each gorilla (i.e., did this include observations from all three contexts). | We used the term “cumulative eigenvector centrality” as that is what was used in the papers we modeled this analysis after (Bret et al., 2013; Ramos et al. 2019), but agree it is not 100% clear.  To clarify, this term is better described as the “reverse EC rank” such that a gorilla who had the largest EC value was ranked 14 (out of 14 gorillas) and the lowest EC value was ranked 1 (out of 14 gorillas). We have reworded this section to better describe how these values were calculated.  This was calculated for each observation context separately and thus is not a sum of each observation type (as shown in Figure 3).  We clarified these points and the sentence now reads: LINE 270 “To test for the presence of socially central individuals we graphed each individual’s eigenvector centrality against their reverse eigenvector centrality rank (i.e. greatest centrality score was ranked 14) for each observation condition [64,65].” |
| Line 276: The authors consider a modularity score greater than 0.30 to indicate subgrouping as suggested by Whitehead, but is there any reason to believe this threshold is appropriate for gorillas? Is there evidence (for instance from mountain gorillas) that this low modularity score predicts subgroup membership? It would be useful if the authors could provide some additional justification here and whether this value is biologically relevant for gorillas. | This is a fantastic question and one that sent us down the rabbit hole a bit. The short answer is no – there is no specific evidence to suggest this threshold is meaningful for gorillas as no study of gorillas to-date (published) has conducted a similar measure (*one study did but data are not extractable, see further comment below).  Kasper & Voelkl (2009) conducted an interesting study where they compiled data from 70 groups of 30 primate species (including gorillas). They then applied a variety of common social network analysis procedures on these data (but do not provide species specific outcomes) and described each measure’s usefulness (or lack thereof) in relation to describing primate social groupings. For modularity (Q) the authors note that an overall mean value was 0.21 (SD 0.16). The authors note the variability with this estimate suggests subgroups can be “clearly identifiable (pg. 349)” but again provide no mathematical reasoning on why any threshold value should demarcate subgrouping. While this also provides no specific reasoning as to why this metric could apply to gorillas, they do state “If the subgroup assignment is not based on individual characteristics but on a modularity-maximizing algorithm, then one should ask for the biological interpretation of this partitioning (pg. 349).” This then emphasizes Q may itself not be a defining measure of modularity without proper consideration of the practicalities of what is being described (i.e. does the subgrouping make some sort of sense or have some clear pattern associated with it that can be used to describe why there is modularity).  With all of this in mind, we:   - Provided additional context to the methods explaining the uncertainty with gorillas. This now reads LINE 284 “To-date, no work has clarified if a Q value ≥ 0.30 is biologically relevant for gorillas, thus based on the recommendations of Kasper & Voelkl [69] any Q value ≥ 0.30 was followed up with additional analysis to detect if biologically relevant factors were correlated with the clusters. For this follow up analysis a Dietz’ R-test was used to determine whether subgroup membership was defined by shared origin or sex.” - We additionally add this point to the discussion of subgroupings. LINE 535 “Though the subgroupings here do not appear to be random, it is again worth mentioning that the quantitative threshold for identifying subgroupings here is not based on gorilla sociality itself, but rather broad mathematical predictions [68]. From a biological perspective it is unlikely that the same modularity score applied to two different species has the same interpretive value, thus we echo points raised by Kasper & Voelkl [69] that modularity scores themselves should be interpreted cautiously.” |
| Line 284: In Figure 1 it appears as if the size of these nodes is quite similar. Does this indicate that the sum of individual associations was identical for every gorilla? This does not appear to be the case based on edge weights. This would need to be clarified or the difference in node size needs to be made more apparent. | Yes the nodes do appear similar - though they are different - but we agree it is not visually obvious. We regenerated the network graphs using the Gephi software rather than SOCPROG as the latter was limited in its ability to adjust scaling parameters. We have updated our visualization methods description in the method section beginning LINE 294 to reflect these changes and have submitted an updated version of Figure 1. |
| Line 304: It is a bit unnecessary to list out the specific numbers for dyads here as these are arbitrary based on how the order of dyads were listed in the association matrix. Would be better to rephrase to “three gorilla dyads had an association rate greater…” | The line here specifies that 36 pairs, 21 pairs and 26 pairs had association rates greater than the group mean – not that only three pairs (labeled by those #’s) had above mean values. We have reworded this to be clearer. Now reads: LINE 316 “Association rates were greater than the group mean for 39.6% (36/91), 23.1% (21/91), and 28.6% (26/91) of dyads in the mixing, nesting and shifting contexts, respectively, suggesting there was a level of selectiveness in how the gorillas chose to associate” |
| Lines 398 – 400: It is intriguing that the females in this group appear to form stronger associations with each other than those observed in the wild. Is there any indication from other captive primate populations that birth control implants (if used) have any effect on female-female interactions? This could be another reason for the observed strength of association among the GRACE females. Without having pre- and post-implant behavioral data, it would be hard to rule this out. | BC and associations in non-human primates is understudied but absolutely a possibility. We have added this to the discussion:  LINE 423 “An additional factor of living in human care that may influence these gorillas’ behavior is the use of oral contraceptives. In humans, the use of contraceptives have been associated with a wide range of behavioral changes [76], including influences on social relationships [77]. Data from a single zoo-living gorilla group suggests the influence of contraceptives on female gorilla behavior may be limited [72]. Data from other primate taxa are also limited and have reported varying levels of influence on behavior [78]. This makes drawing firm conclusions on their influence within our study challenging, however, these drugs remain a potential confounding variable in making comparisons to populations in nature.” |
| Lines 423 – 428: In addition to the fact that these individuals are in captivity, it is also notable that all the females within this group were at or near the age of reproduction for this species (as noted in Table 1) during the observation period in 2022. In the wild, the high centrality of adult male gorillas is often a function of the close affiliative associations maintained with weaned, younger offspring. This distinction in group age structure cannot be ignored and should be noted here in the discussion. | Thank you for this suggestion.  We have added on discussion to this point in the paragraph that reads: LINE 545 “One notable difference between mountain gorilla silverbacks and the GRACE silverback is that the social centrality of mountain gorillas is partially supported by their relationships with immature individuals [4]. At GRACE, the silverback maintained a similarly central position despite the group only containing reproductive aged females and maturing males. It may be that in the absence of immature gorillas adult females associate more with the silverback (or vice versa) at GRACE increasing his social centrality, though more study is needed to fully contextualize this difference.” |
| Conclusions: The authors note in Lines 114 and 118 that the sanctuary’s ultimate goal will be to release gorillas into the wild. It would be beneficial for the authors to indicate (either in the discussion or conclusion) how the observations in this study on dyad-specific associations might influence management decisions. For instance, how might long-term affiliations among specific dyads affect their outcomes if released into the wild? | Thank you for this important suggestion.  Per similar recommendations from reviewer 1 we have expanded on this point in the introduction (see line numbers above) and have added content to the discussion – see LINE 431. |
